# Quality indicators of palliative care for cardiovascular intensive care

**DOI:** 10.1186/s40560-022-00607-6

**Published:** 2022-03-14

**Authors:** Yoshimitsu Takaoka, Yasuhiro Hamatani, Tatsuhiro Shibata, Shogo Oishi, Akemi Utsunomiya, Fujimi Kawai, Nobuyuki Komiyama, Atsushi Mizuno

**Affiliations:** 1 Department of Cardiovascular Medicine, St. Luke’s International Hospital, 9-1, Akashi-cho, Chuo-ku, Tokyo, Japan; 2 Department of Cardiology, National Hospital Organization Kyoto Medical Center, Kyoto, Japan; 3 Division of Cardiovascular Medicine, Department of Internal Medicine, Kurume University School of Medicine, Fukuoka, Japan; 4 Division of Cardiovascular Medicine, Hyogo Brain and Heart Center, Himeji, Japan; 5 Department of Critical Care Nursing, Human Health Sciences, Kyoto University Graduate School of Medicine, Kyoto, Japan; 6 St. Luke’s International University Library, Tokyo, Japan; 7 Penn Medicine Nudge Unit, University of Pennsylvania Philadelphia, Philadelphia, PA USA; 8 Leonard Davis Institute for Health Economics, University of Pennsylvania, Philadelphia, PA USA; 9 Division of Cardiovascular Medicine, Hospital of the University of Pennsylvania, Philadelphia, PA USA

**Keywords:** Palliative care, Cardiovascular disease, Intensive care, End-of-life, Domains, Quality indicators

## Abstract

Healthcare providers working for cardiovascular intensive care often face challenges and they play an essential role in palliative care and end-of-life care because of the high mortality rates in the cardiac intensive care unit. Unfortunately, there are several barriers to integrating palliative care, cardiovascular care, and intensive care. The main reasons are as follows: cardiovascular disease-specific trajectories differ from cancer, there is uncertainty associated with treatments and diagnoses, aggressive treatments are necessary for symptom relief, and there is ethical dilemma regarding withholding and withdrawal of life-sustaining therapy. Quality indicators that can iterate the minimum requirements of each medical discipline could be used to overcome these barriers and effectively practice palliative care in cardiovascular intensive care. Unfortunately, there are no specific quality indicators for palliative care in cardiovascular intensive care. A few indicators and their domains are useful for understanding current palliative care in cardiovascular intensive care. Among them, several domains, such as symptom palliation, patient- and family-centered decision-making, continuity of care, and support for health care providers that are particularly important in cardiovascular intensive care.

Historically, the motivation for using quality indicators is to summarize mechanisms for external accountability and verification, and formative mechanisms for quality improvement. Practically, when using quality indicators, it is necessary to check structural indicators in each healthcare service line, screen palliative care at the first visit, and integrate palliative care teams with other professionals. Finally, we would like to state that quality indicators in cardiovascular intensive care could be useful as an educational tool for practicing palliative care, understanding the minimum requirements, and as a basic structure for future discussions.

**Supplementary Information:**

The online version contains supplementary material available at 10.1186/s40560-022-00607-6.

## Introduction

Cardiovascular disease is one of the major causes of deaths worldwide. In the past several decades, in addition to the progress of surgical procedures and less-invasive devices, the birth of cardiac intensive care units (CICUs) has contributed to the outcome improvement in cardiovascular disease patients [1]. The role of CICUs has shifted from care for patients with acute coronary syndrome without complications to more complex patients, including heart failure with extracardiac organ dysfunction, high-risk pulmonary embolism, malignant arrhythmia, acute aortic syndrome, and cardiogenic shock, especially necessary for monitoring [2–6]. Even with the progress of the treatment strategies, the mortality rate in the CICUs is still high compared to other general wards due to the complex nature of background characteristics [2]. Therefore, healthcare providers working in CICUs should be competent in dealing with patients’ death and end-of-life care [7]. In the past several decades, the perception and attitude toward death has dramatically changed according to the aging society, both for the patients and the physicians. For example, the concept of “less is more” has been spreading around the world [8]. This concept raises the issue of overtreatment, and it is becoming increasingly important to ensure that the level of care provided matches that of patients, families, and society. Considering this background, palliative care, which usually manages patients’ death and end-of-life care, has gained more attention in the field of cardiovascular intensive care [9].

**Fig. 1:**
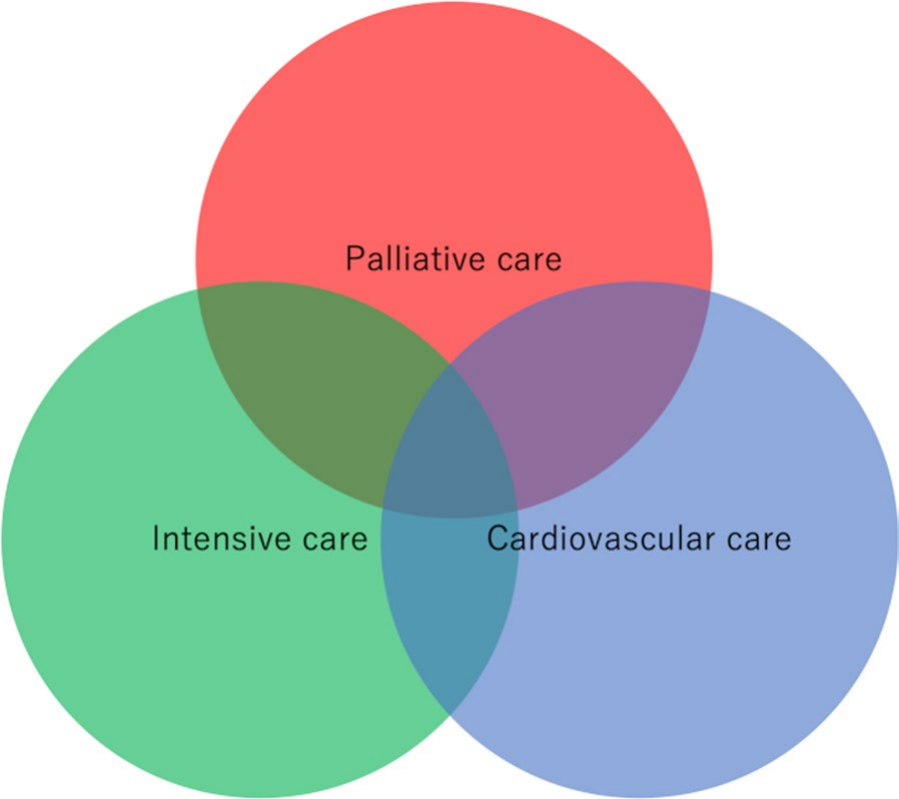
Scope of cardiovascular intensive care unit. The fields of palliative care, intensive care, and cardiovascular care overlap

Several barriers to the integration of palliative care and critical care have long been discussed and guidelines for cardiovascular palliative care have been recently published [10–12]. However, palliative care in the field of cardiovascular intensive care, CICUs, and the integration of these three specialties: palliative care, cardiovascular care, and intensive care, are still in its infancy (Fig. 1). In this article, we focus on palliative care in the field of cardiovascular intensive care and introduce the quality indicators for acute cardiovascular disease, which might be useful for many healthcare providers to implement palliative care in cardiovascular intensive care, as well as for educational purposes.

## The definition of palliative care and several barriers in implementing it in cardiovascular intensive care

The term “palliative care” is popular; however, what “palliative care” indicates is vague [13]. Palliative care is usually defined by the World Health Organization as “An approach that improves the quality of life of patients and their families facing problems associated with life-threatening illness, through prevention and relief of suffering by means of early identification, impeccable assessment, and treatment of pain and other problems: physical, psychological and spiritual” [14]. Although many physicians totally agree with this common definition and the general concept of palliative care, we could not differentiate “palliative care” from “medicine itself, which are based on similar concepts [15]. Due to broadening of the spectrum of palliative care from cancer to chronic disease, palliative care is largely affected by external social needs. The attitude to tackle patients’ unmet needs, in other words “problematization” is the fundamental concept of palliative care [15]. Although the term “palliative care” is often mistakenly regarded identical to “end-of-life care, many palliative care specialists often declare that the provision of palliative care should depend on need not prognosis [16]. However, this needs-driven palliative care model sometimes faces difficulties, especially due to resource limitation, such as access to palliative care team [17]. For example, one-third of providers do consider daily participation in intensive care unit rounds by the palliative care team as an optimal way [18]. We should balance the patients’ need and available resources in each hospital and each intensive care unit.

Palliative care has been developed mainly in the field of cancer patients and its coverage has expanded to many non-cancer patients [19]. In addition to the complexity of palliative care itself, there are several challenges and barriers to its implementation in the field of cardiovascular disease compared to that of cancer. First, the disease trajectory differs between patients with cancer and patients with cardiovascular disease. It is well known that the trajectory of cardiovascular disease is characterized by an overall gradual decline in function with intermittent serious episodes and exacerbations, which is in contrast to that of cancer with a short period of evident decline [20]. Especially in the phase of serious episodes, such as admission to the intensive care unit, not only patients but also physicians are not sure whether the patients could recover to the status before admission. This uncertainty related to cardiovascular disease makes palliative care strategy difficult to apply [21]. A survey of palliative care in the intensive care unit revealed that patients and their families often express unrealistic expectations, which could result in a barrier to integrating palliative care into intensive care [18]. Second, the treatment for baseline conditions differ from that of cancer patients. Symptom palliation is one of the important elements in palliative care, and many symptoms overlap between cancer and cardiovascular disease patients [22]. However, one important aspect of symptom palliation in cardiovascular disease is that guideline-based treatments for cardiovascular diseases, such as vasodilators or inotropes, could be effectively used [23]. For example, dyspnea is a frequently observed symptom in patients with cardiovascular disease. Because guideline-based optimization of conventional therapies, such as angiotensin-converting enzyme inhibitors, can ameliorate dyspnea, we should refrain from blinded opioid prescription before that [23]. These treatment differences would result in a more aggressive strategy in the end-of-life phase of cardiovascular disease compared with cancer patients and the exact same palliative care strategy would not be applicable in the field of cardiovascular disease [24]. Of course, refractory physical symptoms should be managed by a palliative care specialist, but this should be done alongside fundamental treatments for heart failure itself [25]. Finally, cardiovascular disease could be associated with unstable hemodynamics, including cardiopulmonary arrest, which could be restored by life-sustaining therapy including cardiopulmonary resuscitation (CPR) and mechanical circulatory support devices. Life-sustaining therapy can prolong patients’ lives; however, it is sometimes not desirable for patients and their families. In addition to these fundamental CPRs, the discussion about withdrawing and withholding these mechanical circulatory devices in the end-of-life phase is quite important but still challenging. For example, venoarterial extracorporeal membrane oxygenation (VA-ECMO) provides temporary oxygenation and perfusion to patients with cardiopulmonary failure [26]. Compared with potential long-term usage of mechanical ventilation, VA-ECMO has no long-term option, owing to the technology itself. For the patient who fails to recover and are not eligible for transplantation or destination therapy with ventricular assist devices, there is only terminal discontinuation. Although continuing life-sustaining therapy for prolonged period itself could be the goal of care for patient’s family, treating physicians generally need surrogate consent to withdraw life-sustaining therapy, including VA-ECMO. This can present a dilemma and end-of-life conflicts between healthcare providers and families, leaving physicians wanting a greater degree of professional autonomy [27]. As destination therapy using ventricular assist device was approved and reimbursed this May 2021 in Japan, these new options could make it more difficult for treating physicians to obtain surrogate consent to withdraw life-sustaining therapy.

## The quality indicators for palliative care field

Considering these difficulties of palliative care for cardiovascular disease, the detailed components of palliative care might need to be clarified for many clinicians to implement it in cardiovascular intensive care effectively. Although some guidelines or statements described components of palliative care narratively, iterations or checklists would be better implemented [11, 28]. Quality indicators are one of the major options for iterating minimum requirements for each medical field. Quality of care itself is defined as “the degree to which health services for individuals and populations increase the likelihood of desired health outcomes and are consistent with current professional knowledge” [29]. One of the definitions of quality indicator is “quantitative measures that provide information about the effectiveness, safety and/or people-centeredness of care.” Across several definitions of quality indicators, there are three essential components: (1) quality goal, (2) measurement concept, and (3) appraisal concept. Quality indicators are also sometimes classified into several categories [30]. Among these, the most widely used classification of quality indicators in healthcare, Donabedian’s Structure–Process–Outcome (SPO) framework proposed by Donabedian, included three levels of class: structure, process, and outcome of care [31]. Briefly, the structure is applicable to the environment the instruments for palliative care, such as the presence of a palliative care team and 24/7 access to the palliative care team. Process is the actual medical treatment and care provided. Outcome is usually considered as actual outcome, such as mortality of each target population, but in palliative care context, bereaved family survey was only available form as an indicator of quality. The National Consensus Project and National Quality Forum provided eight major domains to capture palliative care: (1) structure and process of care; (2) physical aspects of care; (3) psychological and psychiatric aspects of care; (4) cultural aspects of care; (5) spiritual, religious, and existential aspects of care; (6) ethical and legal aspects of care; (7) care of the patient at the end of life; (8) social aspects of care. [32] In the field of cancer, several quality indicators for palliative care have already been discussed and updated according to these domains [33–35]. From the perspective of quality improvement measuring, quality of end-of-life care, palliative care utilization and site of death have been discussed in the palliative care for cancer patients [36]. As for quality of end-of-life care, many aggressive treatments especially during end-of-life period were frequently discussed and monitored. For example, proportion receiving chemotherapy in the last 14 days of life and admission to the ICU in the last month of life were frequently monitored. According to the claim data, the proposed appropriate threshold for proportion receiving chemotherapy in the last 14 days of life and admission to the ICU in the last month of life were 10% and 4%, respectively [37]. Palliative care utilization and site of death have also been frequently discussed especially based on the whether the medical care was consistent with the patient’s needs [38, 39]. Not only would the indicators themselves but also the strategy and frameworks to make quality indicators be useful for many non-cancer field healthcare providers, especially in CICUs, to understand the current concepts and methods of palliative care. In the following sections, we will introduce detailed examples and lists of quality indicators of palliative care in intensive care and cardiovascular intensive care.

## Quality indicators and domains of palliative care in intensive care

There are only a limited number of studies on quality indicators for palliative care in intensive care units (not limited to cardiovascular disease). Clarke et al. summarized 53 quality indicators and seven domains: (1) patient- and family-centered decision-making, (2) communication, (3) continuity of care, (4) emotional and practical support, (5) symptom management and comfort care, (6) spiritual support, and (7) emotional and organizational support for intensive care unit clinicians [40]. These seven domains largely overlap with the eight domains of general quality indicators for palliative care by NCP and NQF as described above. Patient- and family-centered decision-making, continuity of care, and support for health care providers are not covered by NCP and NQF domains and could be considered as a unique point of quality indicators for palliative care in the intensive care unit [41]. Metaxa et al. performed a systematic review of palliative intervention in the intensive care unit field and reported that these seven domains were not practical, because many of them overlapped with each other [42]. Half of interventions are categorized into patient- and family-centered decision-making and one-third are categorized into communication within the team and with patients and families. Therefore, they advocated a more pragmatic classification of palliative intervention in the intensive care unit by following the intervention taxonomy framework, which summarizes palliative interventions as follows: (1) communication interventions, (2) ethics consultations, (3) educational interventions, (4) palliative care team involvement, and (5) advance care planning [42]. Using these five new categories, half of them are categorized into palliative care team intervention. Both of these, the five new intervention categories and seven domains by Clarke et al., are not perfect, but would be useful to comprehend the current important aspects of palliative care in cardiovascular intensive care.

Studies on quality indicators of palliative care for cardiovascular intensive care are scarce. We performed an updated and a structured PubMed literature review on quality indicators for palliative care in cardiovascular disease patients, with only two articles related to cardiovascular intensive care (Additional file 1). We contrasted these two studies with NQF eight domains and Clarke’s most popular quality indicator of palliative care in intensive care unit, as shown in Table 1. In addition, details of each quality indicators are shown in Additional file 2: Table S1. Hamatani et al. listed 35 quality indicators for palliative care in patients with heart problems and Mizuno et al. made 21 quality indicators for acute cardiovascular disease [43, 44]. The indicators for heart failure included appropriate heart failure treatment and care, which is similar to performance measures for heart failure treatment itself [45]. These indicators implied that even if we consider palliative care for patients with heart problems, we should not forget baseline treatment as described above. Hamatani et al. also measured these indicators in patients with heart problems from three teaching hospitals, which revealed that three indicators were quite low performance: “intervention by multidisciplinary team,” “opioid therapy for patients with refractory dyspnea,” and “screening for psychological symptoms.” The other palliative care quality indicator for acute cardiovascular disease mainly focuses on palliative care itself. The two major domains were "symptom palliation" and "supporting the decision-making process.” The seven sub-categories included: “presence of palliative care team”, “patient–family relationship”, “multidisciplinary team approach”, “policy of approaching patients”, “symptom screening and management”, “presence of ethical review board”, “collecting and providing information for decision-maker”, and “determination of treatment strategy and the sharing of the care team’s decision”.

**Table 1:** Comparison of domains about quality indicators for palliative care

Clarke et al. [34]	National Quality Forum [35]	Hamatani et al. [37]	Mizuno et al. [38]
Patient and family-centered decision making	Structure and processes of care	Structure and process of disease care	Presence of palliative care team
Communication within the team and with patients and families	Physical aspects of care	Appropriate HF treatment and care	Patient family relationship
Continuity of care	Psychological and psychiatric aspects of care	Total pain management	Multidisciplinary team approach
Emotional and practical support for patients and families	Physical aspects of care	Decision support and ethical issue management	Policy to approach patients
Symptom management and comfort care	Spiritual, religious, and existential aspects of care		Symptom screening and management
Spiritual support for patients and families	Ethical and legal aspects of care		Presence of ethical committee
Emotional and organizational support for intensive care unit clinicians	Care of the patient at the end of life		Collecting and providing information for decision-maker
	Social aspects of care		Determination of treatment strategy and the sharing of their decision
			Outcome measures

## Symptom palliation and support decision-making process

**Fig. 2:**
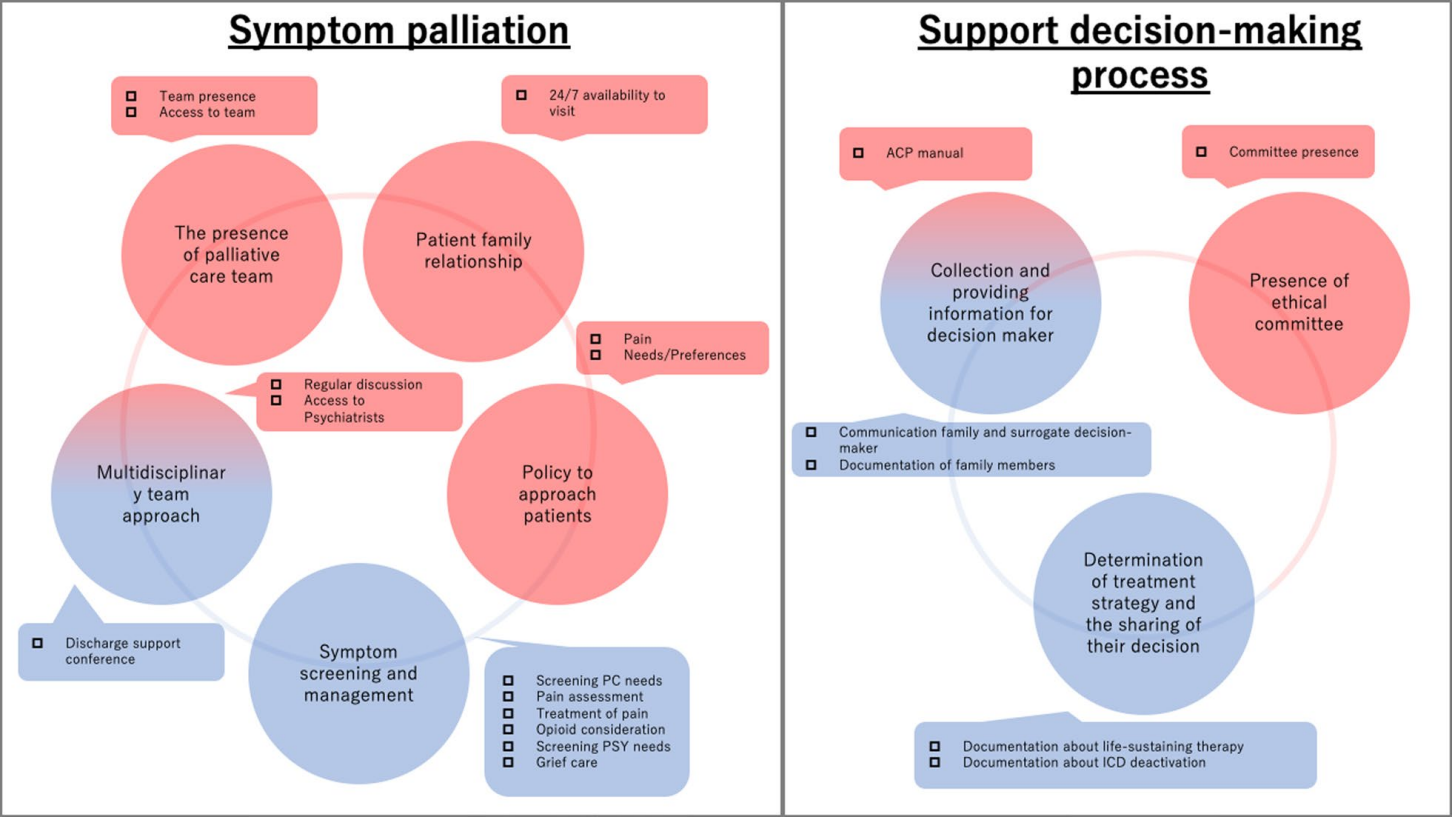
Quality indicators in acute cardiovascular disease. Symptom palliation and support decision-making are major domain. The symptom palliation domain includes five subdomains and the support decision-making domain includes three subdomains. Red components indicated the structural indicators. Blue components indicated process indictors. *PC* palliative care, *PSY* psychiatric symptom, *ACP* advanced care–planning, *ICD* implantable cardioverter defibrillators

Symptom palliation and support decision-making processes are two major domains of quality indicators of palliative care in cardiovascular intensive care and reflect structural and process indicators in the SPO framework (Fig. 2). The symptom palliation domain includes five subdomains. These domains and clinical indicators are based on the concept “total pain,” which was advocated by Cicely Saunders and consisted of physical, psychological, social, and spiritual pain [46]. Each clinical indicator embraced total pain concept and indicated that we should effectively and efficiently screen total pain and ameliorate these spectra of pain. Based on these quality indicators, there are three steps to approach the total pain. Although palliative care interventions to improve patients’ symptoms in the chronic phase have been evaluated in the past several years, there is no specific randomized control trial to evaluate the impact of palliative care on cardiovascular intensive care. In addition, even in the chronic phase, the impact of palliative care interventions remains inconsistent. Palliative care in heart failure (PAL-HF) was the first randomized, controlled clinical trial reported in 2017, which evaluated the additional palliative care intervention on usual care in patients with heart problems [47]. Rogers et al. reported that palliative care intervention improved disease-specific quality of life evaluated by the Kansas City Cardiomyopathy Questionnaire, depression, anxiety, and spiritual well-being. Sahlollbey et al. recently performed a systematic review and meta-analysis of palliative care on patient outcomes, which revealed that palliative care intervention could improve total symptom burden measured by the Edmonton Symptom Assessment Scale (standardized mean difference − 0.29; 95% CI − 0.54 to − 0.03) [48]. They also reported that there are only three studies evaluating symptom burden, and for individual symptoms, there was no clear impact of palliative care on anxiety, dyspnea, or pain. Quinn et al. also performed a systematic review and meta-analysis of palliative care interventions in chronic noncancer illness [49]. They reported that palliative care was significantly associated with lower symptom burden translated to the Edmonton Symptom Assessment Scale (standardized mean difference − 1.6; 95% CI − 2.6 to − 0.4). Finally, regarding symptom palliation, we should pay attention not only to patients’ symptoms but also to caregivers’ symptoms at the same time. Shinada et al. reported that about 15% of caregivers experienced depression and complicated grief after the death of a patient due to acute cardiovascular disease, which was also encompassed by symptom palliation of quality indicators [50].

The support decision-making process is imperative for current palliative care in cardiovascular intensive care. As described above, decision-making in cardiovascular intensive care is more complex and time limited compared to other healthcare settings. There are two important essences of supporting the decision-making process [51]. First, we should determine the surrogate decision-maker with whom we can discuss patients’ treatment strategy. Ideally, we should discuss this with the patients themselves. However, in cardiovascular intensive care settings, patients often cannot communicate with healthcare providers due to loss of consciousness or cognitive dysfunction [52]. In this situation, we should determine and document who should be approached about the patients’ care or carefully discuss with patients and surrogate decision makers through advance care planning [53]. Of note, any decision making should be toward the patient himself. Second, we should acknowledge that many decisions making in cardiovascular intensive care depends not on the best available evidence but on patients and their families’ preferences, which is associated with ethical dilemmas. Furthermore, not only patients but also healthcare providers cannot predict patient prognosis, which results in decision-making under uncertainty. Conquering these dilemmas, several ethical topics should be prepared in the field of cardiovascular intensive care, including withholding and withdrawal of the treatment, especially life-sustaining therapy and terminal/palliative sedation. A previous systematic review revealed that most of the recommendations referred to withholding and withdrawal of life-sustaining therapy, and these two were considered as morally equivalent and permissible [54]. Although both withholding and withdrawal of life-sustaining therapy have increased recently, withholding of treatment is perceived as less difficult even if the end result is the same. The difficulty in withdrawing life-sustaining therapy is often explained by a status-quo bias. The decisions to maintain the status quo tend to be regretted less than decisions to change, which could best apply to the status, where the patient has already been intubated and needs to discuss the withdrawal [55]. A surrogate decision-maker should be encouraged to share the decision with others that could reduce their responsibility and minimize the risk of being blamed. Finally, continuous deep sedation, which is sometimes referred to as terminal or palliative sedation, is also an important ethical and a sensitive topic. Previous cardiovascular intensive care quality indicators did not include this specific topic; however, it is important for many clinical professionals. Continuous deep sedation could sometimes be confused with euthanasia by non-healthcare professionals, which could result in understanding gaps between healthcare professionals and non-healthcare professionals [56]. We should recognize that the intent and documentation of the intent of physicians and other healthcare providers are important after careful discussions.

Finally, as described above, many patients and families frequently do not have any experiences about advance care planning, lack medical background knowledge, and are under time-restricted situation in intensive care unit. To support this, several decision aids and programs support surrogate decision maker have been developed [57–59]. As the effectiveness of these educational materials and programs are still controversial [60], future trials would be necessary to implement patient and family side education for an ideal decision making. More importantly, getting familiar with these ethical topics and noticing the presence of ethical dilemmas could be essential for implementing palliative care for cardiovascular intensive care.

## How to use quality indicators of palliative care for cardiovascular intensive care

Theoretically, there are two major motivations for using quality indicators. The first is for a quality assurance system as a summative mechanism for external accountability and verification [61]. Pay for quality of care, such as pay for performance programs, is categorized into this. The second is a formative mechanism to improve quality. Internal audit and feedback for continuous improvement at the hospital level can be categorized into a formative mechanism. For quality assurance and accountability, high-level precision and advanced statistical techniques are required; otherwise, providers will resist the usage of the quality indicator itself and its potential consequences, such as certification issues. Unfortunately, there is still no sufficient evidence and high-quality randomized control trials to prove the validity and importance of quality indicators in cardiovascular intensive care. Even in the cancer field, many quality indicators of palliative care have not been well accepted for accountability purposes, which implies that many quality metrics are too difficult to monitor or do not directly reflect the quality of care in each department and hospital. Only a few of them, mainly structure indicators, such as specialist allocation, are valid and easily measured.

**Fig. 3:**
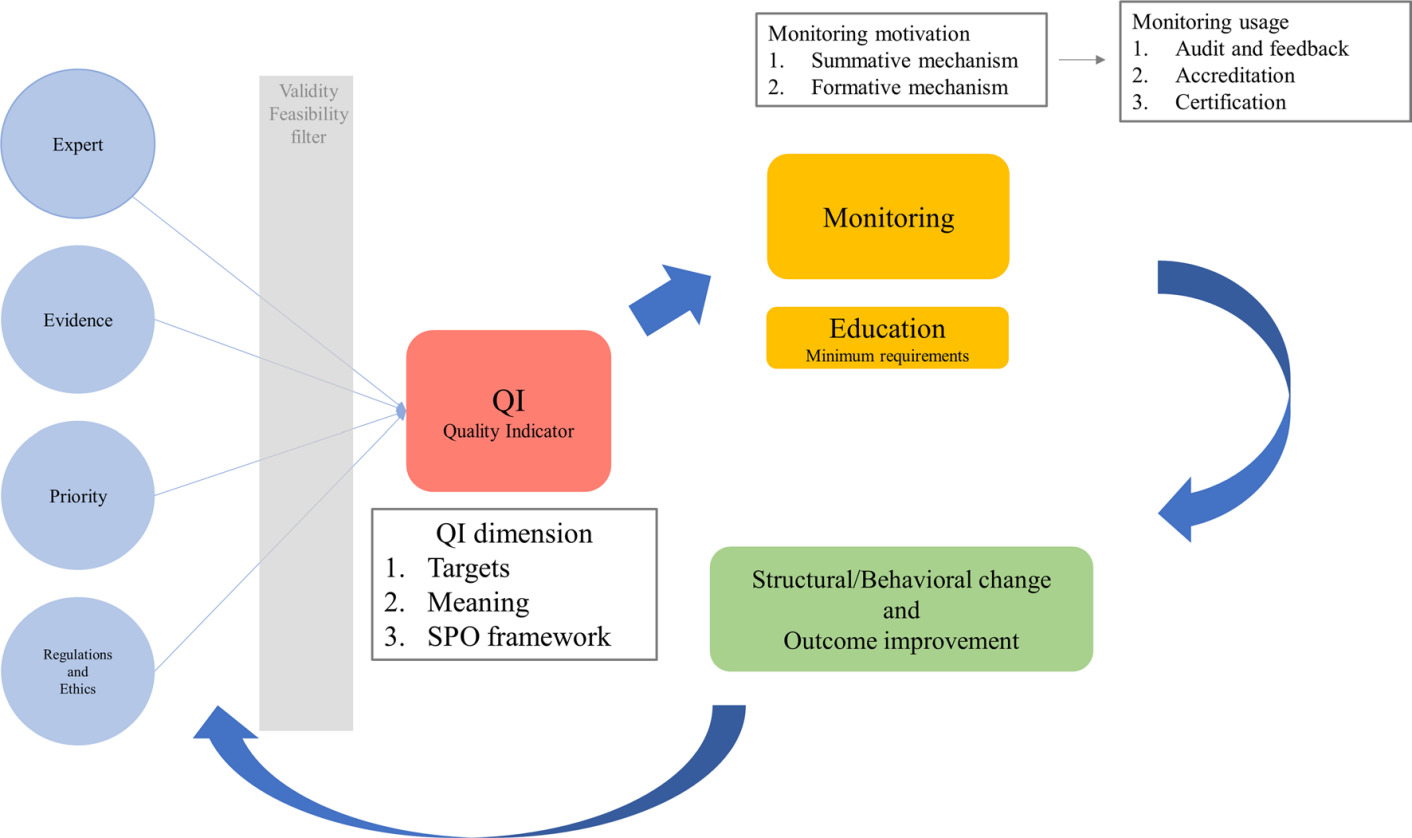
Scheme of development and usage of quality indicators. Quality indicators are based on expert opinion, evidence, political priorities, regulations, and ethical positions. In addition, quality indicators are educational tools to help people understand the nature of a particular field. Quality indicators are also used to monitor the quality of care in hospitals and departments. Structural/behavioral changes after monitoring and education lead to quality improvement. These quality improvement processes are a continuous and iterative cycle. *SPO* Structure–Process–Outcome

Considering these usage limitations in palliative care quality indicators for quality assurance, some other insights are needed to use quality indicators (Fig. 3). First, it is important to know that the quality indicators are derived not only from the evidence but also from expert opinions, policy priorities, regulations, and ethical standpoints. Quality indicators made in each target disease or field and the domain sorted by the SPO framework are usually useful for understanding the essence of a specific field. After recognizing these backgrounds, as discussed, quality indicators and domains would be useful for learning the minimum requirements for quality improvement. Especially in cardiovascular intensive care, palliative care is yet to be considered a common practice, and could be useful for many beginners to understand the minimum requirements for palliative care in the field of cardiovascular intensive care. Furthermore, after monitoring each quality indicator, structural and behavioral changes are necessary to improve palliative care quality. There are several barriers to implementing palliative care, especially in intensive care units. Design modifications or behavioral scientific approaches will be necessary to modify the structure and care process in the intensive care unit [62]. Using behavioral insights and implementation strategies would be helpful to tackle the evidence–practice and quality indicator–practice gap [63]. These processes of making quality indicators and using quality indicators for quality improvement are continuous and repetitive cycles similar to the Plan–Do–Check–Action cycle to create new quality indicators.

Finally, we should acknowledge that there are several limitations in the use of quality indicators and speculations on how to use indicators effectively. As described above, quality indicators of palliative care in cardiovascular intensive care have not been fully evaluated and have not been validated with patient outcomes. The domain and subcategories could also be just hypothetical contrasts, and we could not deny arbitrariness. The following strategies could be realistically implemented to use quality indicators of palliative care for cardiovascular intensive care from a clinical perspective. First, we should check the structure indicators, including workflow, policy, and resources about palliative care in each healthcare service line, such as hospitals and departments. Second, palliative care needs and symptoms should be screened at the first encounter and then repeatedly. Finally, we should integrate multidisciplinary palliative care teams and other specialists when necessary, mainly reflected by process indicators.

## Conclusion

Although there are limited numbers and limited usage of quality indicators of palliative care for cardiovascular intensive care compared with other fields, quality indicators could be used as educational tools to implement palliative care and the fundamental structure for future discussion. To implement palliative care for cardiovascular intensive care, we should learn several basic concepts of palliative care through quality indicators and monitor indicators to see, where we stand now. With the increasing demand for palliative care for every patient, further high-quality evidence and valid quality indicators are warranted.

## Supplementary Information

**Additional file 1.** Initial search for quality indicators of palliative care in heart disease.

**Additional file 2: Table S1.** Comparison of quality indicators for palliative care.

## Data Availability

Not applicable.
