# Supplementary material for: Quality indicators of palliative care for cardiovascular intensive care
Source: J Intensive Care. 2022 Mar 14;10:15. doi: 10.1186/s40560-022-00607-6 (PMC8922808; doi:10.1186/s40560-022-00607-6)
Supplement: Supplementary file 1 — Additional file 1. Initial search for quality indicators of palliative care in heart disease. [file 40560_2022_607_MOESM1_ESM.docx]

**Additional file on search strategies**

***Initial search for quality indicators of palliative care in heart disease***

We updated a previous structured PubMed literature review on quality indicators for palliative care in acute cardiovascular disease patients on 30th October 2021 using the following search terms: ("quality indicators, health care"[MeSH Terms] OR "quality indicator*"[Title/Abstract] OR "quality measure*"[Title/Abstract] OR "clinical indicator*"[Title/Abstract] OR (("quality"[Title] OR "performance"[Title] OR "satisf*"[Title]) AND ("indicator*"[Text Word] OR "criteri*"[Text Word] OR "assess*"[Title] OR "measur*"[Title] OR "scale"[Title] OR "validat*"[Text Word]))) AND ("cardiovascular diseases"[MeSH Terms] OR ("heart"[Title] OR "cardiac"[Title]) OR "cardiovascular"[Text Word]) AND ("palliative medicine"[MeSH Terms] OR "hospice and palliative care nursing"[MeSH Terms] OR "hospices"[MeSH Terms] OR ("terminal"[Text Word] OR "hospice*"[Text Word] OR "palliative"[Text Word] OR "end of life"[Title/Abstract]) OR "terminal care"[MeSH Terms] OR "Life Support Care"[MeSH Terms] OR "advance care planning"[MeSH Terms] OR "withholding treatment"[MeSH Terms] OR "terminally ill"[MeSH Terms] OR "palliative care"[MeSH Terms]) AND 2015/01/01:2021/12/31[Date - Publication].[^1^](#_ENREF_1)^,^[^2^](#_ENREF_2)^)^ We found 94 publications. Unfortunately, after reviewed by two authors (A.M., Y.T.) with clinical backgrounds in cardiology, these are only two publications were eligible as studies including quality indicators about palliative care for cardiovascular intensive care.[^3^](#_ENREF_3)^)^ As described in the main manuscript, we contrasted these two studies with NQF eight domains and Clarke’s most popular domains and quality indicators of palliative care in intensive care unit (Table 1 and Additional Table S1).[^4^](#_ENREF_4)^,^[^5^](#_ENREF_5)^)^

**References**

1 Kamal AH, Gradison M, Maguire JM. et al. Quality measures for palliative care in patients with cancer: a systematic review. Journal of Oncology Practice 2014:JOP. 2013.001212.

2 Mizuno A, Miyashita M, Hayashi A. et al. Potential palliative care quality indicators in heart disease patients: A review of the literature. J Cardiol 2017;70:335-41.

3 Pasman HRW, Brandt HE, Deliens L. et al. Quality indicators for palliative care: a systematic review. Journal of pain and symptom management 2009;38:145-56. e21.

4 Clarke EB, Curtis JR, Luce JM. et al. Quality indicators for end-of-life care in the intensive care unit. Crit Care Med 2003;31:2255-62.

5 Ferrell B, Connor SR, Cordes A. et al. The national agenda for quality palliative care: the National Consensus Project and the National Quality Forum. J Pain Symptom Manage 2007;33:737-44.
