# Supplementary material for: Quality indicators of palliative care for cardiovascular intensive care
Source: J Intensive Care. 2022 Mar 14;10:15. doi: 10.1186/s40560-022-00607-6 (PMC8922808; doi:10.1186/s40560-022-00607-6)
Supplement: Supplementary file 2 — Additional file 2: Table S1. Comparison of quality indicators for palliative care. [file 40560_2022_607_MOESM2_ESM.docx]

Additional Table S1. Comparison of quality indicators for palliative care

| **Clarke EB et al. Quality indicators for end-of-life care in the intensive care unit. Number of measures, 53** |
| --- |
| **Patient and family-centered decision making** |
| Recognize the patient and family as the unit of care |
| Assess the patient’s and family’s decision-making style and preferences |
| Address conflicts in decision making within the family |
| Assess, together with appropriate clinical consultants, the patient’s capacity to participate in decision making about treatment and document assessment |
| Initiate advance care planning with the patient and family |
| Identify the healthcare proxy or surrogate decision maker |
| Clarify and document resuscitation orders |
| Assure patients and families that decision making by the healthcare team will incorporate their preferences |
| Follow ethical and legal guidelines for patients who lack both capacity and a surrogate decision maker |
| Establish and document clear, realistic, and appropriate goals of care in consultation with the patient and family |
| Help the patient and family assess the benefits and burdens of alternative treatment choices as the patient’s condition changes |
| Forgo life-sustaining treatments in a way that ensures patient and family preferences are elicited and respected |
| **Communication within the team and with patients and families** |
| Meet as interdisciplinary team to discuss the patient’s condition, clarify goals of treatment, and identify the patient’s and family’s needs and |
| Address conflicts among the clinical team before meeting with the patient and/or family |
| Utilize expert clinical, ethical, and spiritual consultants when appropriate |
| Recognize the adaptations in communication strategy required for patients and families according to the chronic vs. acute nature of illness, cultural and spiritual differences, and other influences |
| Meet with the patient and/or family on a regular basis to review patient’s status and to answer questions |
| Communicate all information to the patient and family, including distressing news, in a clear, sensitive, unhurried manner, and in an appropriate setting |
| Clarify the patient’s and family’s understanding of the patients’ condition and goals of care at the beginning and end of each meeting |
| Designate primary clinical liaison(s) who will communicate with the family daily |
| Identify a family member who will serve as the contact person for the family |
| Prepare the patient and family for the dying process |
| **Continuity of care** |
| Maximize continuity of care across clinicians, consultants, and settings |
| Orient new clinicians regarding the patient and family status |
| Prepare the patient and/or family for a change of clinician(s) and introduce new clinicians |
| **Emotional and practical support for patients and families** |
| Elicit and attend to the needs of the dying person and his/her family |
| Distribute written material (booklet) for families that includes orientation to the ICU environment and open visitation guidelines, logistical information (nearby by hotels, banks, restaurants, directions), listings of financial consultation services, and bereavement programs and resources |
| Facilitate strengthening of patient-family relationships and communication |
| Maximize privacy for the patient and family |
| Value and support the patient’s and family’s cultural traditions |
| Arrange for social support for patients without family or friends |
| Distribute written material (booklet) containing essential logistical information and listings of financial consultation services and bereavement support programs/resources |
| Support the family through the patient’s death and their bereavement |
| **Symptom management and comfort care** |
| Emphasize the comprehensive comfort care that will be provided to the patient rather than the removal of life-sustaining treatments |
| Institute and use uniform quantitative symptom assessment scales appropriate for communicative and noncommunicative patients on a routine basis |
| Standardize and follow best clinical practices for symptom management |
| Use nonpharmacologic as well as pharmacologic measures to maximize comfort as appropriate and desired by the patient and family |
| Reassess and document symptoms following interventions |
| Know and follow best clinical practices for withdrawing life-sustaining treatments to avoid patient and family distress |
| Eliminate unnecessary tests and procedures (laboratory work, weights, routine vital signs) and only maintain intravenous catheters for symptom management in situations in which life-support is being withdrawn |
| Minimize noxious stimuli (monitors, strong lights) |
| Attend to patient’s appearance and hygiene |
| Ensure family’s and/or clinician’s presence so the patient is not dying alone |
| **Spiritual support for patients and families** |
| Assess and document spiritual needs of the patient and family on an on-going basis |
| Encourage access to spiritual resources |
| Elicit and facilitate spiritual and cultural practices that the patient and family find comforting |
| **Emotional and organizational support for ICU clinicians** |
| Support healthcare team colleagues caring for dying patients |
| Adjust nursing staff and medical rotation schedules to maximize continuity of care providers for dying patients |
| Communicate regularly with interdisciplinary team regarding goals of care |
| Establish a staff support group, based on the input and needs of ICU staff and experienced group facilitators, and integrate meeting times into the routine of the ICU |
| Enlist palliative care experts, pastoral care representatives, and other consultants to teach and model aspects of EOLC |
| Facilitate rituals for the staff to mark the death of patients |
| **National Quality Forum. Palliative and End-of-Life Care off-Cycle Measure Review 2017. Number of measures, 36** |
| **Physical aspects of care** |
| Improvement in Pain Interfering with Activity |
| Comfortable Dying: Pain Brought to a Comfortable Level Within 48 Hours of Initial Assessment |
| Oncology: Plan of Care for Pain – Medical Oncology and Radiation Oncology (paired with 0384) |
| Oncology: Medical and Radiation - Pain Intensity Quantified (paired with 0383) |
| Pain Assessment and Follow-Up |
| Percent of Residents Who Self-Report Moderate to Severe Pain (Short-Stay) |
| Percent of Residents Who Self-Report Moderate to Severe Pain (Long-Stay) |
| Patients Treated with an Opioid who are Given a Bowel Regimen |
| Patients with Advanced Cancer Screened for Pain at Outpatient Visits |
| Hospice and Palliative Care — Pain Screening |
| Hospice and Palliative Care — Pain Assessment |
| Hospice and Palliative Care — Dyspnea Treatment |
| Hospice and Palliative Care — Dyspnea Screening |
| External Beam Radiotherapy for Bone Metastases |
| **Psychological and psychiatric aspects of care** |
| Health-related Quality of Life in COPD patients before and after Pulmonary Rehabilitation |
| **Cultural aspects of care** |
| Cross-Cultural Communication Measure Derived from the Cross-Cultural Communication Domain of the C-CAT |
| **Spiritual, religious, and existential aspects of care** |
| Beliefs and Values - Percentage of Hospice Patients with Documentation in the Clinical Record of a Discussion of Spiritual/Religious Concerns or Documentation That the Patient/Caregiver Did Not Want to Discuss |
| **Ethical and legal aspects of care** |
| Advance Care Plan |
| Patients Admitted to ICU who Have Care Preferences Documented |
| Hospice and Palliative Care – Treatment Preferences |
| **Care of the patient at the end of life** |
| Family Evaluation of Hospice Care |
| Proportion Receiving Chemotherapy in the Last 14 Days of Life |
| Proportion Admitted to the ICU in the Last 30 Days of Life |
| Proportion Not Admitted to Hospice |
| Proportion Admitted to Hospice for Less Than 3 Days |
| Bereaved Family Survey |
| Hospitalized Patients Who Die an Expected Death with an ICD that Has Been Deactivated |
| CAHPS Hospice Survey (Experience with Care): 8 PRO-PMs: (Hospice Team Communication; Getting Timely Care; Getting Emotional and Religious Support; Getting Hospice Training; Rating of the Hospice Care; Willingness to Recommend the Hospice; Treating Family Member with Respect; Getting Help for Symptoms) |
| Hospice and Palliative Care Composite Process Measure—Comprehensive Assessment at Admission |
| **Hamatani et al. Development and Practical Test of Quality Indicators for Palliative Care in Patients with Chronic Heart Failure. Number of measures, 35** |
| **Structure and process of disease care** |
| Presence of multidisciplinary team |
| Availability of multidisciplinary team |
| Regular discussion by multidisciplinary team |
| Intervention by multidisciplinary team |
| **Appropriate HF treatment and care** |
| Consideration of β-blocker prescription |
| Consideration of ACEI/ ARB prescription |
| Consideration of MRA prescription |
| Explanation of ICD therapy |
| Explanation of CRT therapy |
| Consideration of cardiac transplantation |
| Evaluation of CAD and valvular heart disease |
| Education for secondary prevention |
| Consultation service for ICD implantation |
| **Total pain management** |
| Preparation of screening sheet for total pain |
| Description of goals of total pain management |
| Symptom evaluation using quantitative scales |
| Management of physical pain |
| Preparation of opioid instruction |
| Opioid therapy for patients with refractory dyspnea |
| Evaluation of constipation during opioid therapy |
| Evaluation of nausea and vomiting during opioid therapy |
| Screening for psychological symptoms |
| Availability of psychiatrist |
| Grief care for family members |
| Survey of family structure |
| Conferencing for discharge support |
| Consideration of withholding or withdrawing life- prolonging treatment |
| Multidisciplinary discussion about ICD deactivation at the end of life |
| ICD deactivation prior to death |
| Multidisciplinary team discussion about palliative sedation |
| Informed consent of palliative sedation |
| **Decision support and ethical issue management** |
| Preparation of instruction for the illness trajectory of HF |
| Preparation of medical manual on advance care planning |
| Multidisciplinary team discussion of life- prolonging treatment |
| Advisory committee for consultation of ethical issues |
| **Mizuno et al. Quality indicators of palliative care for acute cardiovascular diseases. Number of measures, 21** |
| **Presence of palliative care team** |
| The presence of a multidisciplinary palliative care team consisted of cardiologist, physician finishing primary palliative care training, nurse, and pharmacologist. |
| Access to the multidisciplinary palliative care team on weekdays |
| **Patient family relationship** |
| 24/7 availability for families to visit patients in intensive care unit |
| **Multidisciplinary team approach** |
| Regular discussion (at least once a week) by multidisciplinary team, and charting |
| Access to psychiatrists in cases of alarming psychiatric symptoms including delirium, are suspected |
| Discharge support conference before discharge |
| **Policy to approach patients** |
| The hospital has measures (e.g., questionnaire and templates) to evaluate patients’ needs and preferences |
| The hospital has a policy on how to measure and treat pain |
| **Symptom screening and management** |
| Assessment and documentation of pain in patients with acute cardiovascular diseases within 24 h after admission |
| Screening of palliative team intervention in acute cardiovascular diseases |
| Pharmacological/Non-pharmacological treatment for patients with strong pain (such as numerical rating scale !4) |
| Consideration given to opioid prescription for patients with refractory dyspnea |
| Screening for psychiatric symptoms |
| Grief care planning for bereaved family members before patient’s death |
| **Presence of ethical committee** |
| There is an ethical committee that manages ethical dilemmas such as conflict of preferences, and difficulties in determining treatment strategy |
| **Collecting and providing information for decision-maker** |
| Manual for healthcare providers on advanced care-planning |
| Communication with family and surrogate decision-makers within 24 h after admission |
| Documentation for family members, relatives, and surrogate decision- makers |
| **Determination of treatment strategy and the sharing of their decision** |
| Documentation of discussions with patients and their families about the cessation/withholding of life-sustaining therapy including cardiopulmonary resuscitation in acute cardiovascular disease patients (no time limitation) |
| Documentation about the deactivation of ICD |
| **Outcome measures** |
| Bereaved family survey |
